# Prolonged high Myl9 levels are associated with the pathogenesis and respiratory symptom of post-acute COVID-19 syndrome: A 6-month follow-up study

**DOI:** 10.1016/j.clinsp.2025.100584

**Published:** 2025-01-28

**Authors:** Jun Sugihara, Chiaki Iwamura, Tomoya Tateishi, Tadashi Hosoya, Sho Shimada, Kiyoshi Hirahara, Shinsuke Yasuda, Yasunari Miyazaki

**Affiliations:** a Department of Respiratory Medicine, Graduate School of Medical and Dental Sciences, Institute of Science Tokyo, Tokyo, Japan; b Department of Immunology, Graduate School of Medicine, Chiba University, Chiba, Japan; c Department of Rheumatology, Graduate School of Medical and Dental Sciences, Institute of Science Tokyo, Tokyo, Japan

**Keywords:** Post-acute COVID-19 syndrome, Myosin Light Chain 9, Respiratory Symptoms, Neutrophil

## Abstract

**Background:**

Post-acute COVID-19 Syndrome (PACS) occurs in some COVID-19 patients long after acute infection and significantly affects patients’ health. However, the mechanism by which PACS develops is unknown. Myosin light chain 9 (Myl9), produced by activated platelets, plays a role in immune dysregulation and microthrombi formation during acute COVID-19. However, in the PACS phase, the association between Myl9 and residual symptoms remains unclear, and further investigation is needed.

**Methods:**

In this prospective cohort study, serum Myl9 concentrations were measured in 195 COVID-19 patients during hospitalization and at 3- and 6-month follow-up visits. Gaussian mixture modeling was used to identify groups on the basis of Myl9 levels. Relationships between Myl9 levels and residual symptoms were evaluated. Clinical characteristics influencing Myl9 levels were analyzed via logistic regression.

**Results:**

A total of 304 serum samples from 195 patients were collected. Two distinct groups were identified in the Myl9 distribution with a cutoff of 386 ng/mL by Gaussian mixture modeling in this cohort. The high-Myl9 group presented significant residual respiratory symptoms at 6 months post-infection (*p* < 0.05). Elevated Myl9 levels at 6 months were correlated with increased neutrophil counts (*p* < 0.01) and respiratory comorbidities at diagnosis (*p* < 0.05) according to univariate regression analysis. Multivariate regression analysis confirmed the relationship between the neutrophil count and high Myl9 levels.

**Conclusion:**

Prolonged high Myl9 levels are associated with respiratory symptoms, suggesting the potential involvement of prolonged inflammation or endothelial damage in PACS.

## Highlights

• Respiratory symptoms of PACS are associated with high serum Myl9 levels.
• Patients who have had COVID-19 can be divided into two groups according to their serum Myl9 levels.
• The neutrophil count is correlated with the Myl9 level at 6 months after infection.

## Introduction

COVID-19 is an emerging infectious disease that has caused a worldwide pandemic. To date, over 760 million cases of infection and 7 million related deaths have been reported globally.^1^ Whereas some patients recover from acute respiratory infection, other patients experience long-term residual symptoms.^2^ This condition is called Post-Acute COVID-19 syndrome (PACS) and occurs in approximately 50 % of patients >6 months after acute infection.^3^, ^4^, ^5^ PACS is related to poor health-related quality of life^6^^,^^7^; Therefore, it is crucial to understand the mechanism underlying PACS and how to prevent its occurrence.

Myl9 is a small molecular protein that serves as a regulatory light chain of non-muscle myosin 2A, which participates in cell movement processes such as cell division, adhesion, and migration.^8^ Moreover, Myl9, a ligand for CD69, is involved in various chronic inflammatory diseases, such as allergic airway inflammation, inflammatory bowel disease, and Kawasaki disease.^9^^,^^11^ In the case of SARS-CoV-2 infection, high concentrations of plasma Myl9 have been detected, especially in severe cases.^12^ Moreover, microthrombi, which characterize in pathological changes in fatal cases of COVID-19, contain Myl9.^12^ These findings indicate that Myl9 is associated with dysregulated immunity and microthrombi formation in the acute phase of COVID-19.

These two pathologic phenomena are postulated to be involved in PACS^13^^,^^14^; therefore, Myl9 may also play a role in PACS. However, the former study tracked plasma Myl9 only in the acute phase of COVID-19; thus, it is not clear how Myl9 changes over time and whether Myl9 is associated with PACS.

In line with these findings, the authors measured Myl9 in a post-acute COVID-19 cohort and investigated its dynamics and relationship with PACS. In this study, the authors aimed to determine the involvement of Myl9 in PACS and to elucidate certain aspects of its pathophysiology.

## Materials and methods

### Study design and patients

This was a prospective cohort study. The authors enrolled patients who were admitted to Tokyo Medical and Dental University Hospital for the treatment of COVID-19 from Apr 1st, 2020, to Oct 31st, 2021, and who attended follow-up visits. The authors excluded patients who were under 20 years old or declined to participate in the study.

The patients’ diagnoses of COVID-19 were confirmed with positive results on reverse-transcription PCR assays for SARS-CoV-2.

For this study, the authors assembled two cohorts: 1) Patients who had available serum samples collected during hospitalization, at the 3-month visit, or at the 6-month visit were included in cohort 1; 2) Patients in cohort 1 who had 6-month clinical records, laboratory data and serum samples were included in cohort 2.

### Data collection

The authors collected clinical findings such as sex, age, height, body weight, smoking status, comorbidities at onset, extent of respiratory failure in the acute phase, medications used in the acute phase, and residual or emerging symptoms due to COVID-19 at the 6-month visit. These findings were collected from electronic medical records.

The authors also collected laboratory data at admission, at the 3-month visit, and at the 6-month visit. A complete blood count with white blood cell differential, C-Reactive Protein (CRP), ferritin, and D-dimer were included in the analysis.

### Measurement of serum Myl9

The authors measured the level of Myl9 in serum samples from cohort 1. The patient sera were isolated and stored in two ways. For sera obtained during hospitalization, the authors utilized samples stored at the Bioresource Research Center of the Institute of Science Tokyo. The patient's whole blood was centrifuged at 1700 × g for 10 min at −4 °C, and the serum supernatant was dispensed into a 2 mL microtube and stored at −150 °C until subsequent analyses. For sera obtained at the 3-month or 6-month visit, the authors separated the samples according to the following protocol: patients’ whole blood was centrifuged at 1600 × g for 10 min at room temperature, and the supernatant was dispensed into a 2 mL microtube and stored at −20 °C until subsequent analyses.

The serum Myl9 level was measured via ELISA (human MLC2/MYL9 ELISA kit; LifeSpan BioSciences, Inc., WA, USA) according to the manufacturer's instructions. In brief, serum samples were diluted 1:100 or 1:1000 and applied to 96-well strip plates coated with capture antibody. Then, a biotinylated detection antibody was added, a streptavidin-HRP complex was added, a TMB substrate was added, and a stop solution was added. An appropriate duration and temperature of incubation were used in each reaction step; aspiration of the liquid and adequate washing were conducted between each reaction step. The optical density was read at a wavelength of 450 nm. Serum samples were serially diluted at ≥2 points. The assay Conductor (C.I.) was blinded to each patient's condition.

### Statistical methods

Categorical variables are expressed herein as numbers with percentages, and continuous variables are expressed as medians with interquartile ranges.

Statistical analyses were conducted with R version 4.3.2 as follows: Fisher's exact test was used to assess the ratios of categorical variables, and hypergeometric distribution was used to estimate the confidence interval of the odds ratio. If variables had three or more categories, the Chi-Square test was applied instead. The Wilcoxon rank-sum test was employed for comparisons of two continuous variables. The Kruskal-Wallis test was used for comparisons among three continuous variables. Spearman's rank correlation coefficient was calculated to evaluate correlations between two continuous variables. Univariate and multivariate logistic regression were applied to analyze binary independent variables; p-values < 0.05 were considered to indicate statistical significance.

### Gaussian mixture modeling

The authors used the R package Mclust (version 6.0.1) for Gaussian mixture modeling. First, the authors applied the Mclust function to all measured Myl9 values and acquired unsupervised clustering. Next, the authors defined a cutoff value, as these clusters were separated exclusively.

### Ethical issues

This study was approved by the Ethics Committee of Tokyo Medical and Dental University (G2020–006; July 28th, 2020). The authors obtained written informed consent from all the patients.

## Results

### Measurement of serum Myl9 and its distribution

**Fig. 1:**
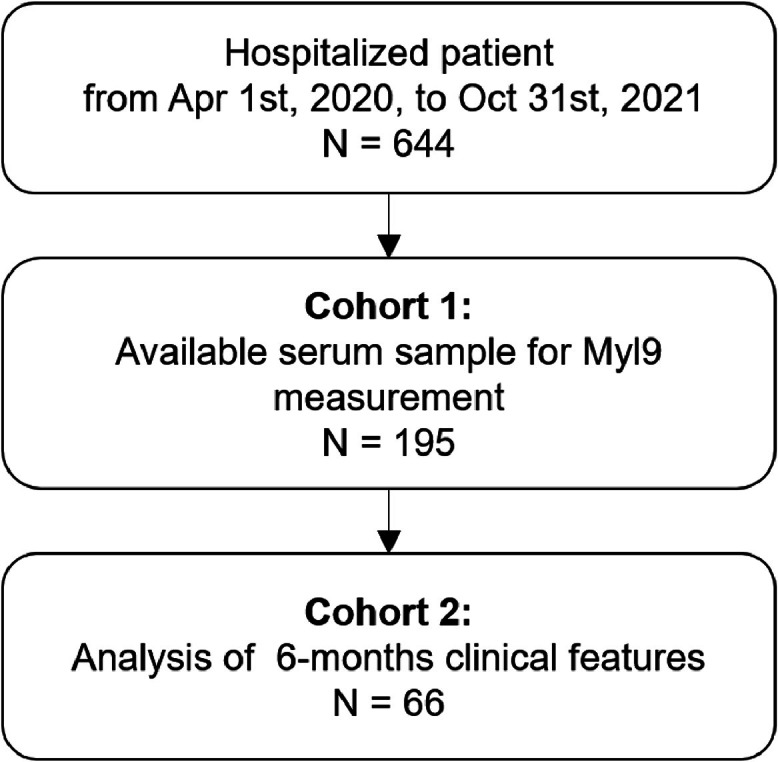
**CONSORT diagram.** Six hundred forty-four patients were hospitalized for COVID-19 during the enrollment period. One hundred ninety-five patients had available serum samples collected during hospitalization, at the 3-month visit, or at the 6-month visit (defined as cohort 1). Sixty-six patients with 6-month clinical records, laboratory data and serum samples were extracted as cohort 2 for further analysis.

The CONSORT diagram is shown in Fig. 1. Among the total of 644 patients who were admitted to the hospital during the study period, 195 patients were included in cohort 1. Patient characteristics are shown in Table 1.

**Table 1:** Patient characteristics.

	**Cohort 1**	**Cohort 2**	**p-value**
**Total number of patients**	195	66	
**Onset date range**	2020/3/29–2021/10/4	2020/3/29–2021/9/20	
**Sex (female)**	70 (35.9 %)	24 (36.4 %)	1.0000
**Age (years)**	56 [47–66]	58 [52–70]	**0.0201**^a^
**(sixty-five or more)**	59 (30.3 %)	28 (42.4 %)	0.0966
**BMI**	24.3 [22.4–27.0]	24.0 [21.9–26.3]	0.5906
**Comorbidity**			
Cardiovascular	24 (12.3 %)	10 (15.2 %)	0.7027
Hypertension	67 (34.4 %)	29 (43.9 %)	0.2122
Diabetes mellitus	31 (15.9 %)	11 (16.7 %)	1.0000
Malignancy (active)	7 (3.6 %)	1 (1.5 %)	0.6657
Malignancy (in remission)	15 (7.7 %)	5 (7.6 %)	1.0000
Autoimmune disease	22 (11.3 %)	11 (16.7 %)	0.3558
Respiratory	28 (14.4 %)	12 (18.2 %)	0.5840
**Smoking status at admission**			0.6551
Never smoker	88 (45.1 %)	27 (40.9 %)	
Ex-smoker	74 (37.9 %)	29 (43.9 %)	
Current smoker	25 (12.8 %)	7 (10.6 %)	
**Respiratory failure in the acute phase**			0.1707
None	72 (36.9 %)	18 (27.3 %)	
Oxygen	101 (51.8 %)	36 (54.5 %)	
Intubation	21 (10.8 %)	12 (18.2 %)	
**Treatment**			
Remdesivir	100 (51.3 %)	33 (50.0 %)	0.9982
Corticosteroid	112 (57.4 %)	43 (65.2 %)	0.3597
Tocilizumab	12 (6.2 %)	5 (7.6 %)	0.9152
Baricitinib	27 (13.8 %)	12 (18.2 %)	0.5553

a Statistically significant p-values (< 0.05).

**Fig. 2:**
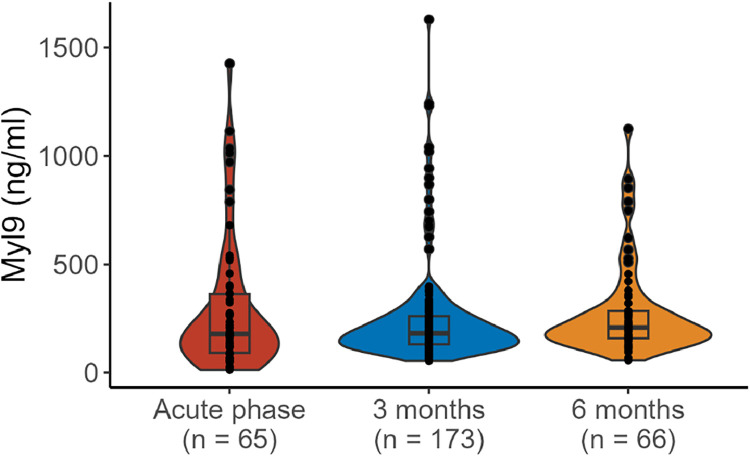
**Serum Myl9 values at each measurement point.** The median values with interquartile ranges were 178.9 [91.6–363.3] ng/mL in the acute phase, 181.7 [131.0–260.9] ng/mL at 3 months, and 208.7 [158.8–280.7] ng/mL at 6 months. The serum Myl9 level at these 3 time points was not significantly different according to the Kruskal-Wallis test (*p* = 0.4776).

In total, the authors analyzed 304 serum samples: 65 samples from the acute phase, 173 samples from the 3-month visit, and 66 samples from the 6-month visit (Fig. 2). The median values with interquartile ranges were 178.9 (91.6–363.3 ng/mL) during hospitalization, 181.7 (131.0–260.9 ng/mL) at 3 months, and 208.7 (158.8–280.7 ng/mL) at 6 months. The serum Myl9 levels at these 3-time points were not significantly different according to the Kruskal-Wallis test (*p* = 0.4776).

**Fig. 3:**
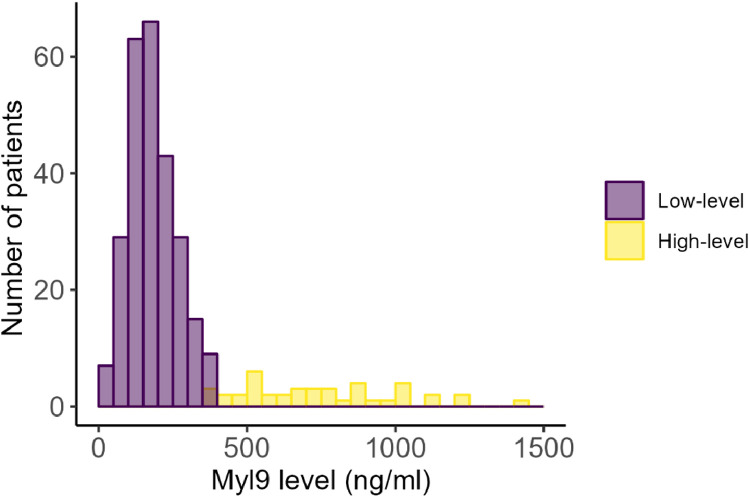
**Distribution of all measured Myl9 values.** The Gaussian mixture model separates these values into two groups: a low-level normally distributed group (purple) and an extended high-level group (yellow). The cutoff point for the two groups was 386 ng/mL.

The distribution of the serum Myl9 values exhibited a single peak with a tail extending toward higher values at all 3 points, as depicted in Fig. 2. To investigate the characteristics of the distribution, the authors applied a Gaussian mixture model. This approach enabled us to elucidate the distribution as a combination of two Gaussian distributions in this post-COVID-19 cohort (Fig. 3). As depicted in Fig. 3, the serum Myl9 levels are divided into two separate Gaussian distributions, which have no overlap in value. On the basis of this analysis, the authors were able to classify patients into two groups; the cutoff level of Myl9 for dividing these two groups was determined to be 386 ng/mL.

### Two groups due to Myl9 levels and the relation to PACS

Next, the authors evaluated the clinical meaning of this grouping. For further analysis, the authors selected 66 patients with 6-month clinical records, laboratory data, and serum samples as cohort 2 (Fig. 1). Among all the clinical findings, only age significantly differed between cohort 1 and cohort 2, and all the other characteristics were regarded as statistically equivalent (Table 1).

**Fig. 4:**
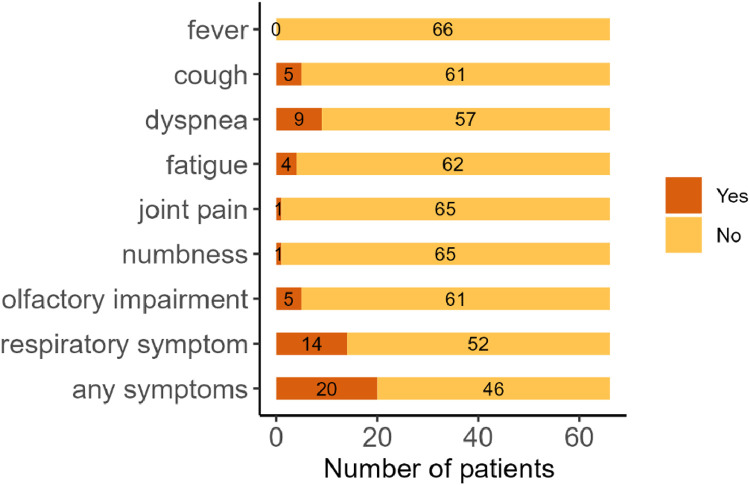
**Number of patients with residual symptoms in cohort 2.** Among the recorded symptoms, fever remained in none of the patients, cough remained in 5 patients (7.6 %), dyspnea remained in 9 patients (13.6 %), fatigue remained in 4 patients (6.0 %), joint pain remained in 1 patient (1.5 %), numbness remained in 1 patient (1.5 %), and olfactory impairment remained in 5 patients (7.7 %). With respect to compositional symptoms, 14 patients (21.2 %) experienced respiratory symptoms, and 20 patients (30.3 %) experienced one or more of these symptoms.

The number of patients with symptoms at 6 months is shown in Fig. 4. The authors aggregated cough and dyspnea into the “respiratory symptoms” category in addition to counting these symptoms separately. Twenty patients (30.3 %) had at least one symptom. Dyspnea was the most common symptom (*n* = 9, 13.6 %), and respiratory symptoms were present in 14 patients (21.2 %).

A comparison of the clinical manifestations of patients in each group on the basis of the Myl9 level revealed that high Myl9 at 6 months was associated with respiratory symptoms at 6 months (Table 2). In addition, as the authors grouped patients who had acute-phase serum according to the cutoff, all patients (*n* = 15) in the high-Myl9 group required supplemental oxygen, whereas 23 of 50 patients in the low-Myl9 group required supplemental oxygen. This difference was statistically significant according to Fisher's exact test (*p* = 0.0001).

**Table 2:** Odds of residual symptoms in each group.

	**Myl9-high**	**Myl9-low**	**Odds ratio [95 % CI]**	**p-value**
**Respiratory symptoms at 6 months**	0.83	0.20	4.26 [1.22–61.3]	**0.0461**^a^
**Any symptoms at 6 months**	0.83	0.38	2.22 [0.62–31.0]	0.2867

a Statistically significant p-values (< 0.05).

### Clinical features accompanying the Myl9 level groups

**Fig. 5:**
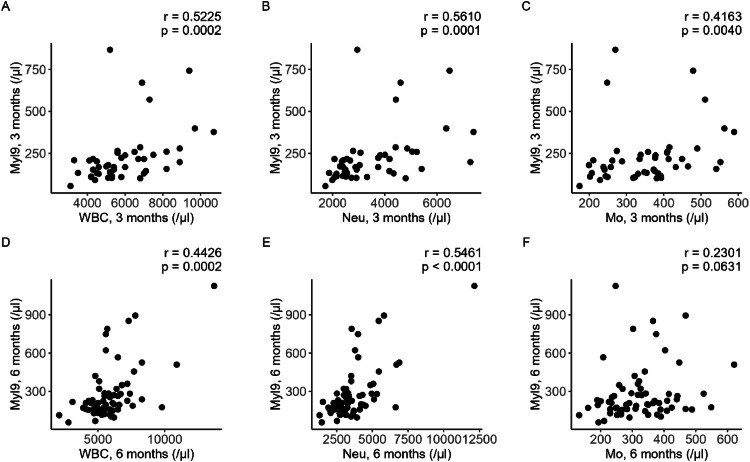
**Correlations between Myl9 values and WBC counts.** Scatter plots of (A) WBC count and Myl9 values at 3-months; (B) Neutrophil counts and Myl9 values at 3 months; (C) Monocyte counts and Myl9 values at 3 months; (D) WBC counts and Myl9 values at 3 months; (E) Neutrophil counts and Myl9 values at 6-months; and (F) monocyte counts and Myl9 values at 6-months. Spearman's rank correlation coefficients (r) and their p-values (p) are depicted.

To elucidate the detailed profiles of high-Myl9 patients at 6 months, the authors explored the clinical characteristics and blood test data of cohort 2. The sequential laboratory data of cohort 2 are summarized in Table 3. The neutrophil count in the peripheral blood and the serum CRP and ferritin levels peaked at the acute phase and then decreased at 3- and 6-months. As shown in Fig. 5, the White Blood Cell (WBC) count and neutrophil count were moderately correlated, and the monocyte number was weakly to moderately correlated with the contemporary Myl9 values.

**Table 3:** Blood test data of cohort 2.

		**Acute phase (*n* = 55)**	**At 3 months (*n* = 58)**	**At 6 months (*n* = 66)**
**WBCs**	(× 10^9^/L)	5.40 [3.95–7.30]	5.60 [4.70–6.90]	5.65 [4.90–6.50]
**Neutrophils**	(× 10^9^/L)	4.17 [2.56–5.87]	2.93 [2.41–4.18]	3.27 [2.59–4.12]
**Lymphocytes**	(× 10^9^/L)	0.99 [0.69–1.24]	1.89 [1.59–2.32]	1.78 [1.30–2.22]
**Monocytes**	(× 10^9^/L)	0.33 [0.24–0.44]	0.35 [0.26–0.41]	0.31 [0.25–0.38]
**Hemoglobin**	(g/L)	144.0 [131.0–151.0]	143.0 [130.0–147.0]	141.5 [131.0–149.0]
**Platelets**	(× 10^9^/L)	191.0 [166.5–250.0]	239.5 [206.0–277.0]	229.5 [195.0–266.0]
**CRP**	(mg/L)	37.9 [9.4–90.6]	0.8 [0.3–1.3]	0.6 [0.3–1.1]
**Ferritin**	(ng/mL)	538 [246–1265]	152 [59–215]	151 [65–231]
**D-dimer**	(mg/L)	0.70 [0.50–1.34]	0.50 [0.50–0.60]	0.50 [0.50–0.74]

WBC, White Blood Cell; CRP, C-Reactive Protein.

** There were missing values for 11 patients in the acute phase and 8 patients at 3 months.

Since the total WBC count and some WBC fractions are related to the serum Myl9 concentration, the authors investigated the associations between the WBC count and the Myl9 level via logistic regression analysis.

In the univariate logistic regression analysis, the predictive factors for the high-Myl9 group included respiratory comorbidities at the time of diagnosis of COVID-19, the WBC count at 3 months, the neutrophil count at 3-months, the monocyte count at 3-months, the WBC count at 6-months, and the neutrophil count at 6-months, as shown in Table 4. These results revealed that the Myl9 level was associated with granulocytes and not with an inflammatory marker, such as CRP, or a coagulation marker, such as D-dimer.

**Table 4:** Univariate logistic regression of the high-Myl9 group.

	**OR**	**95 % CI**	**p-value**
**Sex (female)**	1.0000	[0.2604, 3.8410]	1.0000
**Age (years)**	0.9993	[0.9480, 1.0533]	0.9786
**(sixty-five or more)**	1.1549	[0.3152, 4.2635]	0.8238
**BMI**	1.0321	[0.9116, 1.1686]	0.6179
**Comorbidity**			
Cardiovascular	0.5111	[0.0580, 4.5038]	0.5455
Hypertension	0.6857	[0.1799, 2.6142]	0.5805
Diabetes mellitus	3.9184	[0.9080, 16.9095]	0.0672
Malignancy (active)	N.A.^b^	N.A.^b^	N.A.^b^
Malignancy (in remission)	N.A.^b^	N.A.^b^	N.A.^b^
Autoimmune disease	0.4500	[0.0516, 3.9273]	0.4700
Respiratory	5.7143	[1.3711, 23.822]	**0.0167**^a^
**Smoking history**	1.3879	[0.3617, 5.3265]	0.6328
**Respiratory failure in acute phase**			
Oxygen	4.4737	[0.5297, 37.7849]	0.1687
Intubation	1.9167	[0.4247, 8.6504]	0.3975
**Treatment**			
Remdesivir	1.2000	[0.3266, 4.4085]	0.7836
Corticosteroid	2.7794	[0.5468, 14.1278]	0.2178
Tocilizumab	1.2750	[0.1286, 12.6384]	0.8355
Baricitinib	1.1250	[0.2067, 6.1230]	0.8916
**Laboratory data in acute phase**			
WBCs	1.0001	[0.9999, 1.0002]	0.4422
Neutrophils	1.0001	[0.9999, 1.0002]	0.3718
Lymphocytes	0.9984	[0.9965, 1.0004]	0.1196
Monocytes	1.0009	[0.9979, 1.0040]	0.5616
Hemoglobin	0.7737	[0.5048, 1.1859]	0.2390
Platelets	1.0119	[0.9540, 1.0733]	0.6948
CRP	1.0202	[0.9107, 1.1428]	0.7305
Ferritin	1.0001	[0.9992, 1.0010]	0.8084
D-dimer	1.2130	[0.9358, 1.5724]	0.1447
**Laboratory data at 3 months**			
WBCs	1.0006	[1.0001, 1.0010]	**0.0094**^a^
Neutrophils	1.0007	[1.0002, 1.0012]	**0.0073**^a^
Lymphocytes	0.9998	[0.9986, 1.0011]	0.7733
Monocytes	1.0084	[1.0009, 1.0159]	**0.0272**^a^
Hemoglobin	0.7758	[0.5056, 1.1906]	0.2454
Platelets	1.0279	[0.9391, 1.1251]	0.5507
CRP	1.0396	[0.4120, 2.6233]	0.9345
Ferritin	0.9983	[0.9923, 1.0043]	0.5737
D-dimer	1.2105	[0.9246, 1.5847]	0.1646
**Laboratory data at 6 months**			
WBCs	1.0007	[1.0002, 1.0012]	**0.0069**^a^
Neutrophils	1.0011	[1.0004, 1.0018]	**0.0010**^a^
Lymphocytes	0.9990	[0.9977, 1.0002]	0.1006
Monocytes	1.0059	[0.9992, 1.0125]	0.0824
Hemoglobin	0.9577	[0.6456, 1.4206]	0.8299
Platelets	1.0445	[0.9552, 1.1423]	0.3395
CRP	4.3045	[0.4039, 45.8703]	0.2266
Ferritin	0.9983	[0.9927, 1.0039]	0.5434
D-dimer	1.5631	[0.8478, 2.8817]	0.1524

WBC, White Blood Cell; CRP, C-Reactive Protein.

a Statistically significant p-values (< 0.05).

b Unable to be calculated because of the presence of zero frequency.

**Fig. 6:**
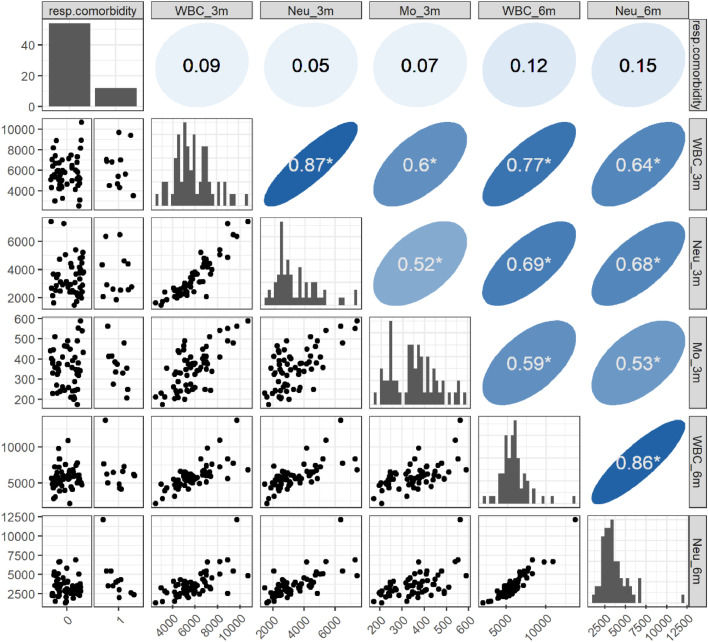
**Correlations among variables detected via univariate logistic regression.** For each pair of variables, scatter plots (lower triangular area) and Spearman's rank correlation coefficients (upper triangular area) are visualized. For respiratory comorbidities, which are binominal variables, dot plots of patients with or without comorbidities (columns 1/0, respectively) are shown instead of scatter plots. The diagonal panels show the distribution of each variable. * Indicates a statistically significant (*p* < 0.05) correlation coefficient. Pairs of WBC, neutrophil, or monocyte counts were significantly correlated with moderate-to-high extents.

Furthermore, the authors performed a multivariate analysis of factors contributing to high Myl9 levels. Before selecting explanatory variables, the authors investigated correlations between variables that were significantly predictive in the univariate analysis and showed strong relationships between WBC, neutrophil, and monocyte counts at 3- and 6-months after onset (Fig. 6). Since including these variables in a model would provoke multicollinearity, the authors chose the neutrophil count at 6-months as a representative variable. The authors also selected respiratory comorbidities for significant correlation with the Myl9 group, age and sex as basic demographic variables, and then constructed a model. As a result, a significant correlation between the neutrophil count and a high Myl9 level was still observed (Table 5), indicating a robust relationship between the two variables.

**Table 5:** Multivariate logistic regression of the high-Myl9 group.

	**OR**	**95 % CI**	**p-value**
**Age**	0.9255	[0.8513, 1.0061]	0.0692^b^
**Sex**	0.6379	[0.0870, 4.679]	0.6583
**Respiratory comorbidity**	6.5510	[0.9306, 46.12]	0.0591^b^
**Neu_6m**	1.0016	[1.0006, 1.0025]	**0.0012**^a^

Neu, Neutrophil count.

a Statistically significant p-values (< 0.05).

b Tendencies (p < 0.1).

## Discussion

In this study, the authors measured the serum Myl9 value during and after COVID-19. The authors previously reported that plasma Myl9 was elevated in the acute phase of COVID-19.^12^ In the present study, the authors confirmed that Myl9 elevation persisted in some patients after the acute phase had passed. The median value of Myl9 in this cohort was approximately 170–200 ng/mL (Fig. 2), which is greater than 39.4 ng/mL, the cutoff value determined by comparing healthy controls and acute COVID-19 patients.^12^ These findings indicate that patients with COVID-19 may experience prolonged inflammation triggered by Myl9 even after they have recovered.

Furthermore, the authors found that patients can be divided into two groups according to their Myl9 levels after contracting COVID-19. Notably, elevated Myl9 levels after COVID-19 are associated with respiratory symptoms, and the blood neutrophil count is correlated with increased Myl9 levels even 6 months after infection.

There were relatively few patients with distinctly elevated Myl9 values, which did not appear to present a normal distribution in this study (Fig. 2). To investigate the distribution of Myl9, the authors employed a Gaussian mixture model, which can interpret the data distribution in its unsupervised nature^15^^,^^16^ and seems appropriate for the nonnormal distribution of the real values of Myl9. This approach revealed that the serum Myl9 levels were divided into two groups: one group followed a normal distribution, whereas the other group presented higher levels beyond the normal range (Fig. 3). This result suggests the existence of multiple phenotypes for Myl9 production or regulation. If a comprehensive examination of factors involved in the regulation of Myl9 is conducted, new insights into the underlying phenotypic differences in the immune system could be obtained in the future.

Another interesting aspect of the study is that the high-Myl9 group had a significantly higher rate of residual respiratory symptoms (Table 2). Previous reports have shown that Myl9 accumulates at inflamed sites and recruits activated CD69-expressing leukocytes, leading to severe and prolonged inflammation.^9^^,^^10^ Since the respiratory system is the central site of inflammation in COVID-19 pneumonia, Myl9 production may occur in the lung and bronchial tissue. Although the pathogenic mechanism of PACS is not fully understood,^13^ it has been proposed that endothelial injury, referred to as “endotheliopathy”, and consequential microthrombosis are probable causes of PACS.^14^ Considering that Myl9 is related to endothelial damage via endothelial inflammation and platelet activation,^17^ the relationship between Myl9 and residual respiratory symptoms may support the “endotheliopathy” hypothesis. Alternatively, higher Myl9 in PACS patients would imply that vasculitis is involved in the occurrence of PACS. Although evidence of vasculitis involvement has not been proposed, some types of SARS-CoV-2 infection manifest as vasculitis.^18^ An increase in the plasma Myl9 concentration has also been confirmed in Kawasaki disease.^11^ These observations support the idea that local vasculitis is one of the causes of pulmonary symptoms in PACS.

The authors also found that patients with at least one respiratory comorbidity, such as bronchial asthma, COPD, or interstitial lung disease, tended to present high Myl9 levels after 6 months of COVID-19 pneumonia, although this difference was not significant according to multivariate regression analysis (Table 5). This result may also be explained by dysregulated inflammation or preexisting damage to the vasculature in individuals with respiratory comorbidities. SARS-CoV-2-induced damage to the vasculature can activate platelets, which produce Myl9 in microthrombi.

In the studied cohort, a higher Myl9 level at 6 months after infection was correlated with a higher blood neutrophil count (Fig. 5). In the acute phase of COVID-19, a correlation between Myl9 levels and neutrophil counts was previously reported.^12^ Myl9 is produced by activated platelets in vessels and enables CD69-expressing leukocytes to attach to a capillary wall and infiltrate inflamed tissue.^9^ Although CD69-expressing leukocytes are generally regarded as activated T-lymphocytes, some neutrophils also express CD69 as a surface marker.^19^^,^^20^ These neutrophils exhibit increased production of TNF-α under combined stimulation with LPS and an anti-CD69 antibody.^19^ Thus, Myl9 may attract and stimulate neutrophils via CD69 and contribute to prolonged tissue damage or inflammation. With respect to the association between residual lung lesions and neutrophils, it has been reported that lung interstitial changes after recovery from acute COVID-19 are related to increased neutrophils in peripheral blood, the proteasome signature in plasma and neutrophil enrichment in cellular deconvolution analyses of RNA from nasal brush samples.^21^ This report implies that systemic and airway neutrophil activation is involved in prolonged pulmonary inflammation, and the present study suggests that Myl9 is a potential connection between pulmonary inflammation and neutrophil activity. To clarify the role of Myl9 and CD69 in neutrophils, future research is needed.

This study has several limitations. First, the single-center nature of the cohort and its limited sample size may have introduced biases. The small number of participants also inhibited a thorough investigation of factors other than Myl9 that may be associated with residual respiratory symptoms. Furthermore, because the enrollment was tailored to patients’ preferences, there may have been selection bias. Nonetheless, the characteristics of the enrolled patients suggest that although the cohort included patients who had undergone hospitalization, it encompassed a range of severities from mild to moderate rather than being skewed toward severe cases. Additionally, the period of patient enrollment resulted in the inclusion of cases from the alpha to delta variants but not from subsequent variants such as omicron. Therefore, different tendencies may be observed in COVID-19 cases caused by later epidemic variants. Additionally, different protocols for storing samples could affect the stability and concentration of Myl9 and potentially lead to inconsistencies in measurement.

## Conclusion

Prolonged elevation of serum Myl9 levels in patients with COVID-19 is associated with respiratory symptoms at 6 months after infection and is strongly correlated with neutrophil counts. These findings suggest that Myl9 may play a role in the pathogenesis of Post-Acute COVID-19 Syndrome (PACS), potentially through prolonged inflammation, endothelial damage, or localized vasculitis. Further research is warranted to explore Myl9 as a potential biomarker and therapeutic target for PACS.

## Declaration of Generative AI and AI-assisted technologies in the writing process

During the preparation of this work, the authors used Deepl translation and chat GPT to find the appropriate expressions, check grammar, and improve the language. After using these services, the authors reviewed and edited the content as needed and took full responsibility for the content of the publication.

## Abbreviations

PACS, Post-Acute COVID-19 Syndrome; Myl9, Myosin light chain 9; CRP, C-Reactive Protein.

## Authors’ contributions

Jun Sugihara: Data curation; formal analysis; visualization; writing-original draft.

Chiaki Iwamura: Formal analysis; investigation; writing-review & editing.

Tomoya Tateishi: Conceptualization; funding acquisition; investigation; project administration; writing-original draft.

Sho Shimada: Data curation; investigation.

Tadashi Hosoya: Conceptualization; writing-review & editing.

Kiyoshi Hirahara: Conceptualization; supervision; writing-review & editing.

Shinsuke Yasuda: Funding acquisition; supervision.

Yasunari Miyazaki: Funding acquisition; supervision.

## Funding

This study was supported by a 10.13039/100019085Japanese Respiratory Foundation grant to T.T. and by the 10.13039/100009619Japan Agency for Medical Research and Development (AMED) under grant number 21ek0410083h0002 to S.Y.

## Declaration of competing interest

The authors declare no conflicts of interest.
